# Corrigendum

**DOI:** 10.1111/pbi.13518

**Published:** 2020-12-28

In the article “Integration of small RNAs, degradome and transcriptome sequencing in hyperaccumulator Sedum alfredii uncovers a complex regulatory network and provides insights into cadmium phytoremediation” by Han *et al*. (2016), the authors would like to correct the SRA database accession number in Materials and Methods section under the sub‐heading sRNA sequencing and miRNAs identification.

The data of SRA database have been transferred to the accession number SRX9346881 as the SRP058333 accession number has been lost.
